# Inflammatory Potential of Diet Is Associated with Biomarkers Levels of Inflammation and Cognitive Function among Postmenopausal Women

**DOI:** 10.3390/nu13072323

**Published:** 2021-07-06

**Authors:** Aleksandra Skoczek-Rubińska, Agata Muzsik-Kazimierska, Agata Chmurzynska, Małgorzata Jamka, Jarosław Walkowiak, Joanna Bajerska

**Affiliations:** 1 Department of Human Nutrition and Dietetics, Poznań University of Life Sciences, Wojska Polskiego 31, 60-624 Poznań, Poland; aleksandra.skoczek-rubinska@up.poznan.pl (A.S.-R.); agata.muzsik@up.poznan.pl (A.M.-K.); agata.chmurzynska@up.poznan.pl (A.C.); 2 Department of Pediatric Gastroenterology and Metabolic Diseases, Poznan University of Medical Sciences, Szpitalna 27/33, 60-572 Poznań, Poland; mjamka@ump.edu.pl (M.J.); jarwalk@ump.edu.pl (J.W.)

**Keywords:** cognitive function, mini-mental state examination, MMSE, dietary inflammatory index, DII, inflammation, menopausal women

## Abstract

In postmenopausal women (PW), estrogen depletion may predispose to cognitive decline through an increased risk of chronic inflammation. Unhealthy diets also appear to have an impact on the cognitive health of these women. The aim of this study was to investigate the association between inflammatory potential of the diet, levels of inflammatory biomarkers, and cognitive function in PW. In a population of 222 PW, energy intake-adjusted Dietary Inflammatory Index (E-DII) was used to assess the dietary inflammatory potential. Cognitive function was estimated using the Polish version of Mini-Mental State Examination (MMSE), corrected by age and educational level. Selected biochemical inflammatory markers (C-reactive protein, CRP; interleukin-6, IL-6; and tumor necrosis factor alpha, TNF-α) were measured by ELISA tests. PW with an anti-inflammatory diet (first tercile) had significantly higher MMSE, while BMI, percentage fat mass and TNFα concentration were significantly lower compared to those with the most proinflammatory diets (third tercile). Women with cognitive impairment had significantly higher IL-6 concentrations (4.1 (0.8) pg/mL vs. 2.5 (0.2) pg/mL, *p* = 0.004), and were less educated (12.7 (0.7) years vs. 14.1 (0.2) years, *p* = 0.03) and less physically active compared to cognitively normal women. PW with the most proinflammatory diets had increased odds of cognitive impairment compared to those with the most anti-inflammatory diets, even after adjustment (OR = 11.10, 95% confidence level; 95%CI: 2.22; 55.56; *p =* 0.002). Each one-point increase in E-DII (as a continuous value) was also associated with 1.55-times greater odds of cognitive impairment (95%Cl: 1.19; 2.02 *p =* 0.003) in this population. Dietary inflammation may increase the risk of cognitive impairment in PW, but future studies should include a more sensitive battery of tests to assess cognitive function in this population. Implementation of an anti-inflammatory dietary pattern in PW may help prevent cognitive decline.

## 1. Introduction

Cognitive impairment is an increasingly pressing public health problem worldwide, with more than one hundred million adults projected to develop dementia by 2050 [1]. Although cognitive impairment does not always reflect incipient dementia, even mild declines in cognitive abilities can cause frustration among people affected by it [1,2]. Accumulating evidence has linked systemic inflammation, as indicated by increased blood levels of circulating proinflammatory cytokines, to decreasing cognitive health and risk of dementia, especially in the later part of life [1]. Systemic inflammation can lead to cognitive decline and dementia by leading the proinflammatory environment in the central nervous system to induce reactive, proinflammatory microglia and astrocytic phenotypes, leading to tau hyperphosphorylation, β-amyloid oligomerization, complement activation, and the breakdown of neurotransmitters into potentially harmful bioactive metabolites [3]. Depletion of estrogen due to menopausal transition is also associated with a decline in cognitive health in women [4,5]. This happens since estrogen benefits hippocampal and prefrontal cortical function, potentially enhancing verbal memory and executive function [6]. Moreover, the depletion of estrogen due to menopause is associated with increasing central obesity. One consequence of this type of obesity is chronic inflammation, which is observed both in the brain and systemically [7]. Au et al. proposed that the relationship between low levels of estrogen and cognitive changes may be mediated by inflammatory processes [6]. However, they concluded that, although the literature supports an interaction between low levels of estrogen, inflammation, and cognitive changes, there is no research to date that could provide empirical support for this hypothesis in postmenopausal women. It has been recognized that lifestyle factors, including unhealthy dietary patterns, appear to play an etiological role in cognitive decline in later life [8]. It has been found that a higher intake of red and processed meat and fried food and a lower intake of whole grains was associated with higher levels of inflammatory markers (interleukin 6 (IL-6), tumor necrosis factor alpha (TNF-α), and C-reactive protein (CRP)), as well as accelerated cognitive decline in older ages [8,9]. To assess the inflammatory potential of diets, the dietary inflammatory index (DII) was developed. Higher DII scores indicate greater inflammatory potential of the diet and have been associated with elevated inflammatory biomarker levels IL-6, CRP, and TNF-α [10]. However, there are limited data on the effect of dietary inflammation on cognitive function in postmenopausal women. We thus hypothesize that women reporting diets with higher proinflammatory potential have higher levels of inflammation markers and perform worse on the Mini-Mental Cognitive Test than women reporting diets with lower inflammatory potential. The objective of this research is to investigate the association between the dietary inflammatory potential circulating levels of inflammatory biomarkers, and cognitive health in a cross-sectional study of midlife women.

## 2. Materials and Methods

### 2.1. Study Population

This study used data from two projects in which postmenopausal women from the Wielkopolska region (Poland) were recruited by newspaper and social media between 2014 and 2020, with similar procedures, including inclusion and exclusion criteria, handling, and storage as have been described elsewhere [11,12]. This study included 222 nonsmoking, naturally postmenopausal women, that is, women without menstruation for at least twelve months, or with serum follicle stimulating hormone (FSH) levels higher than 30 IU/m^2^. We excluded participants who reported unnatural menopause—as a consequence of surgery or radiotherapy for cervix cancer—the use of hormonal replacement therapy (HTZ), or participants who were taking part in weight-loss studies. Women with a history of chronic systemic diseases, such as type 2 diabetes, cardiovascular disease monogenic dyslipidemia, as well as women with serious mental diseases or poor communication skills were not eligible for the current study. Participants who stated that they used hypolipidemic, or hypoglycemic drugs were also excluded from this study. Sociodemographic data, physical activity (PA) data, and cognitive function data were collected through one-to-one interviews with questionnaires, conducted by trained research assistants. The study protocol, as well as the risks and benefits were explained to each woman, and their written consent was received. The local ethics committee at Poznan University of Medical Sciences approved this study (no. 603/14 and 664/20). The study was conducted at the Department of Human Nutrition and Dietetics, Poznań University of Life Sciences.

### 2.2. Anthropometric Measurements

Body weight was measured by plethysmography to the nearest 0.1 kg using a calibrated scale with a Bod Pod system (Cosmed, Italy). Participants were measured after an overnight fast in bathing suits. Height was measured to the nearest 0.1 cm using a stadiometer (RadWag, Poznań, Poland). Body composition was assessed using dual energy X-ray absorptiometry (DXA). All measurements were performed by the same evaluator. The body mass index (BMI) was calculated by the formula: BMI = weight (kg)/[height (m)]^2^.

### 2.3. Assessment of Physical Activity (PA) Level

Physical activity level was determined for all subjects by the short version of the International Physical Activity Questionnaire (IPAQ-SF). The interpretation of this questionnaire assumes a rank of three levels of PA: low level of physical activity (<600 MET/min/week), moderate level of physical activity (from 600 to 1499 MET/min/week), and high level of physical activity (≥1500 MET/min/week).

### 2.4. Assessment of Inflammatory Markers

Blood samples were collected by trained laboratory staff after an overnight fast. Immediately after the sample of venous blood was collected, it was centrifuged and stored at −70 °C for analysis. The concentrations of hs-CRP, TNFα, as well as IL6 were determined in a serum by the enzyme-linked immunosorbent assay (ELISA) method (C-reactive protein; DRG International, NJ, USA; TNF-alpha ELISA, DRG Instruments, Marburg, Germany; Quantikine High sensitivity IL6 ELISA, R&D Systems, Minneapolis, MN, USA) in line with the manufacturer’s directions.

### 2.5. Assessment of Other Covariates

Demographic information, including age, years of education, working situation (as answer to the question “are you currently working, yes or no?”) and living situation (as answer to the question “Do you live alone, yes or no?”) were collected by self-administered questionnaires supported by an individual interview. Details regarding menopausal status, age at menopause, and supplement intake were also obtained.

### 2.6. Dietary Assessment

Dietary intake was estimate by a three-day food diary method in which the participants were trained before the collection of dietary data, with an explanation of what kind of food and beverage intake they should record using household measures. Individuals gave detailed information about the names of food products, herbs and spices used, culinary techniques used, and recipes. Participants also noted whether the food was prepared by themselves at home or purchased. Missing data was additionally obtained during their visits to the Department of Human Nutrition and Dietetics. The food and beverage quantities estimated from the dietary data were converted to grams and milliliters and computed using Dieta 6.0 software (Institute of Food and Nutrition, Warsaw, Poland).

### 2.7. The Dietary Inflammatory Index (DII)

The DII score was calculated to determine the potential inflammatory effect of the diet using the method described by Shivappa et al. [13]. The DII score is calculated using the reported consumption of up to 45 food parameters and in this study, 45 food parameters, derived from mean values of three-day food records, were used: eight proinflammatory nutrients, 19 anti-inflammatory nutrients, ten whole foods and spices, alcohol, caffeine, flavan-3-ol, flavones, flavonols, flavanones, anthocyanidins, and isoflavones. Based on Hayden et al., all 45 food items and nutrients were found to be associated with inflammatory biomarkers (e.g., IL-6, TNF-α, CRP) in older women [10] and scored +1 in the case of dietary factors with proinflammatory effects, −1 for anti-inflammatory dietary items, and 0 for dietary factors that have no effect on the inflammatory biomarkers. This means that a low DII value indicates an anti-inflammatory diet, and a high DII score indicates a proinflammatory diet. Overall, the DII scores in our study ranged from −5.02 (the most anti-inflammatory diet) to 7.92 (the most proinflammatory diet). Energy-adjusted values of DII (E-DII) were calculated per 1000 kcal/day consumed, in order to control for the effect of total energy intake differences between patients.

### 2.8. Cognitive Function Assessment

Cognitive state was examined by the Polish version of the Mini-Mental State Examination (MMSE), the most widely used screening tool for cognitive impairment quantitative assessment. The MMSE is a fully structured scale that consists of thirty points grouped into seven categories: orientation to place, orientation to time, registration, attention, concentration, language, and visual construction [14]. The MMSE test was carried out in accordance with the instructions, taking into account the time limit and the permissible number of three repetitions of each command. The MMSE scale was corrected by age and years of education using the score-adjustment coefficients proposed by Bleecker et al. [15] as follow: MMSE adjusted = MMSE score – (0.471 × (years of education − 12) + (0.131 × (70 − age)). A cut-off score of 25 on the MMSE yielded a sensitivity of 43.3% and a specificity of 90.4% for detecting cognitive impairment [16].

### 2.9. Statistical Analysis

All the statistical analysis was performed using Statistica 13.0 (TIBCO Software, Palo Alto, CA, USA) and PQStat (PQStat Software, Poznań, Poland) software, with the level of significance set at *p* < 0.05. The Shapiro–Wilk test was performed to verify the normality of the distribution of the variables and, as most data were non-normally distributed, nonparametric statistical tests were used. Continuous data are presented as means and standard errors of mean (SEM), and categorical data as *n* (%). To determine the significance among continuous and categorical variables across the distributions of E-DII, the Kruskal–Wallis one-way ANOVA and Chi2 testing, respectively, were performed. Postmenopausal women with cognitive impairment and women with normal cognitive function were compared in the case of continuous and categorical variables using the Mann–Whitney *U*-test and the Chi2 test, respectively. The E-DII scores were divided into three tertiles. Tertile 1 of E-DII below 0.62 included the lowest E-DII scores and presented the most anti-inflammatory group, whereas tertile 3 of E-DII values included the highest E-DII scores over 1.82 and characterized women in the most proinflammatory group. Unadjusted and adjusted multivariable logistic regression models were used to calculate odds ratios and 95% confidence intervals (CI) for the association across the E-DII tertiles and occurrence of cognitive impairment (MMSEadj < 25). Unadjusted and covariate-adjusted linear regression models were also tested to estimate β coefficients and the 95% CI for associations between the continuous variables, E-DII, and cognitive function (MMSEadj score). This statistical analysis used age, years of education, physical activity level, and BMI value as covariates.

## 3. Results

Table 1 shows the characteristics and distributions of women’s cognitive function, anthropometric and biochemical parameters, as well as sociodemographic and lifestyle variables across tertiles of energy-adjusted E-DII scores.

The 222 postmenopausal women enrolled in our study had a mean age of 61. The mean Mini-Mental State Examination score was 27.3 (0.1). The mean BMI value was 31.9 kg/m^2^ (0.4). The mean waist circumference was 103.5 cm (0.8), the mean percentage fat mass was 44.3% (0.4), and mean trunk fat mass was 18.9 kg (0.4). The mean values of hs-CRP, TNFα, and IL-6 was (3.8 mg/L (0.3)), (8.3 pg/mL (0.4)), and (2.7 pg/mL (0.2)), respectively (Table 1). The first tertile characterized women with the highest anti-inflammatory diet (lower E-DII values), and the third tertile was the group of women with the highest proinflammatory diet (higher E-DII values). As was shown in Table 1, MMSEadj was significantly lower, while BMI, percentage fat mass, and TNFα concentration significantly increased across the E-DII score-based tertiles. Hs-CRP and IL-6 increased across the E-DII score-based tertiles, while years of education declined, though insignificantly. Categories of physical activity, living situation, and working status did not differ across E-DII tertiles (Table 1). Moreover, saturated fatty acid (SFA) intake was significantly higher, while polyunsaturated fatty acids (PUFA) and dietary fiber intake significantly decreased across the E-DII score-based tertiles (data not shown).

Table 2 presents the distribution of participants’ cognitive function by E-DII and by anthropometric, sociodemographic, biochemical, and lifestyle variables. Of the total population of 222 women, 25 postmenopausal women (11%) were in the cognitive impairment group and 197 postmenopausal women (89%) had normal cognitive function. Those with cognitive impairment had more proinflammatory diets (2.2 (0.3) vs. 1.1 (0.1), *p* = 0.001), and significantly higher IL-6 concentrations (4.1 (0.8) vs. 2.5 (0.2), *p* = 0.004) than women with normal cognitive function. Women with cognitive problems were also characterized by shorter education than those with normal cognition (12.7 (0.7) vs. 14.1 (0.2), *p* = 0.030). Statistically significant differences (*p* = 0.001) were also noted in women’s physical activity: 41% of women with cognitive impairment reported low levels of physical activity and only 7% reported high levels of physical activity. In the normal cognition group, as many as 75% of women had moderate levels of physical activity and 15% had high levels of physical activity (Table 2). Moreover, women from cognitive impairment group in comparison to normal cognition group had a higher intake of total sugar (40.0 g/day (3.1) vs. 32.1 g/day (1.1); *p* = 0.013) NKT (23.5 g/day (1.8) vs. 20.0 g/day (0.7); *p* = 0.041), and lower intake of dietary fiber (17.2 g/day (1.6) vs. 25.2 g/day (0.8); *p* < 0.001) (data not shown).

Table 3 presents the unadjusted and adjusted odds ratios for cognitive impairment by E-DII tertile and as continuous values of the E-DII score. In the unadjusted model, the postmenopausal women in the highest tertile of E-DII score (most proinflammatory diet) had increased odds of having cognitive impairment than women in the first tertile of DII score (the most anti-inflammatory diet) (OR = 9.05, 95% CI = 2.02; 40.52; P for trend <0.001). This inverse association between E-DII and cognitive impairment remained even after controlling for age, BMI, years of education, and physical activity (adjusted OR = 11.10, 95% CI = 2.22; 55.56; *p* = 0.002). When E-DII was fitted as a continuous variable, each one-point increase in the E-DII score was associated with an increased by a factor of 1.55 in the odds of cognitive impairment (OR = 1.55, 95% CI = 11.19; 2.02; *p* = 0.003).

## 4. Discussion

In this study, postmenopausal women consuming proinflammatory diets had increased odds of having cognitive impairment than those consuming anti-inflammatory diets. The association was strong, even after adjustment for a wide range of potential confounders, including BMI, age, years of education, and physical activity level. We also observed that adherence to the proinflammatory diet was associated with increased levels of TNF-α, confirming the hypothesis that diet modulates inflammation status of postmenopausal women. Moreover, we observed that the postmenopausal women with cognitive impairment had significantly higher E-DII scores and IL-6 levels. These women were also less educated and less physically active. These findings indicate that, apart from the hormonal changes associated with menopause, dietary nutrients with proinflammatory potential may also contribute greatly to worsened cognitive functioning by increasing systemic inflammation. In assessing cognitive functioning, we used the MMSE, which is a popular cognitive assessment instrument frequently utilized in large, population-based cohorts. The mean MMSE score among the postmenopausal women in our study was 27.3 (SEM = 0.1), whereas the mean score on the MMSE among 520 postmenopausal women from Taiwan has been reported as 26.5 (SD = 2.9) [17]. The prevalence of cognitive impairment in our study was 11%, while Sullivan and Woods showed that 62% of women during menopausal transition aged 40–60 had some type of undesirable cognitive changes over the past few years [18]. In their later study performed on a group of 508 postmenopausal women, these authors noted that as many as 72% women reported problems with remembering names and 50% had problems with remembering where they had put items, recent phone numbers, keeping up correspondence, and with forgetting what they were doing a few minutes before [19]. However, in both mentioned studies, memory evaluation was based on women’s perceptions of memory change and their attributions, not on the results of standardized tests for evaluating the cognitive performance of subjects. Indeed, around the time of the menopausal transition, many women report problems with memory and concentration, perhaps suggesting that hormonal changes associated with menopause are linked to memory complaints [4]. However, as was reported in our study and that of Kesse-Guyot et al., a proinflammatory diet in midlife may also be strongly associated with subsequent lower cognitive functioning [20]. Indeed, food items that tend to increase DII scores include butter and other animal fats, as well as sweets. Higher scores for this dietary indicator also result from low consumption of food considered to be anti-inflammatory, such as fruits and vegetables [21,22]. Unfortunately, unhealthy dietary habits are commonly observed among postmenopausal women [12,23]. One explanation given for this is that, in the menopausal state, dysregulation of the hypothalamic appetite control mechanisms occurs, leading to increased consumption of high-fat and high-carbohydrate products [24,25], contributing to inflammation [7]. Indeed, we observed in our study that adherence to a proinflammatory diet was associated with higher levels of TNF-α, and also with nonsignificant increases in CRP and IL-6. It should be noted that, although inflammatory markers are the products of many factors—including age, genetics, and other conditions—our present study aimed to focus on the part of systemic inflammation that can be attributed to nutritional factors, especially diets with proinflammatory potential. Findings linking proinflammatory dietary patterns and inflammation are now growing, and DII has been previously shown to be associated with inflammatory markers in a broad age range of populations, from children [26], through adolescents [27], through the midlife [28], to an older age [21].

In our study we also determined that postmenopausal women with diagnosed cognitive impairment had higher levels of IL-6 and were less educated and less physically active. There are studies suggesting that cytokines circulating in the blood affect the functioning of central nervous system through a variety of pathways [29,30,31]. One of these pathways is direct transport across the blood–brain barrier. The majority of cytokines, including TNFα and, as a rule, IL-6, do not have a saturable bidirectional transport system, and for those cytokines, transportation is only in the blood-to-brain direction [25]. Wright et al. additionally considered that IL-6 may affect cognitive functions through its interactions with vascular health, as there is evidence that higher concentrations of IL-6 increase the risk of diabetes and cardiovascular problems [32]. Bradburn et al., taking into account the results of their meta-analysis, emphasized that high peripheral inflammation measured by blood IL-6 is associated with global cognitive decline in many studies, and should be considered a useful indicator of cognitive health [33]. As in our study, some other pieces of evidence suggest that low education may increase the risk of cognitive disorders [34,35]. Low physical activity is another factor that strongly influences cognitive functioning. For example, the study of Yaffe et al. showed that women with a greater physical activity level at baseline were less likely to experience cognitive decline during six to eight years of follow-up [36].

Some limitations of our study should be mentioned. Firstly, due to the cross-sectional study design, it is difficult to explain the cause–effect relationship of dietary inflammation on cognitive function. Secondly, some of the participants volunteered for an intervention study and may thus not constitute a representative sample of postmenopausal women, as they may be rather focused on receiving help for obesity. Although MMSE is the most widely used tool for screening overall cognitive functioning, it does have some limitation [37]: most importantly, there is a difficulty identifying individuals with cognitive impairment due to the lack of universal cut-offs [38]. Additionally, using only the MMSE to measure overall cognition may have limited our ability to detect some specific cognitive domains related to the menopausal state. The assessment of cognition was also only conducted at one time, so any changes or progress in the cases over time—especially related to severe dementia—could not be captured. More broadly, both socioeconomic problems, such as low wages, and psychological issues, such as depression, can accelerate physical deterioration and cognitive decline.

Our study also has a number of strengths. First, we were able to include forty-five original parameters in the DII calculations, using relatively accurate dietary data. Next, dietary variables not included in the Dieta 6.0 database were supplemented from other sources. As far as we can tell, the studies we looked at in the literature did not always use all 45 dietary variables in calculating DII, so our results have high potential. Our study group is relative homogeneous in terms of other variables affecting cognitive functioning (gender, race, and number of comorbid diseases), which enables easy comparison of the results. Moreover, the MMSE was administered by trained researchers in face-to-face cognitive evaluations, eliminating the possibility that the participants underestimated the questions.

## 5. Conclusions

Our study suggests that proinflammatory diets are associated with an increase in the risk of cognitive impairment in postmenopausal women. However, these results will have to be replicated in other studies using more specific battery tests to assess cognitive function in this population. Furthermore, it may help protect postmenopausal women’s cognitive health to implement an anti-inflammatory diet pattern that includes unprocessed food free of trans-fatty acids, a limited amount of saturated fatty acids, and high levels of polyunsaturated fatty acids, especially omega-3 fatty acids and polyphenols. Such a diet could help reduce the risk of cognitive decline.

## Figures and Tables

**Table 1:** Characteristics and distributions of study participants across tertiles of energy-adjusted dietary inflammatory index (E-DII).

Parameters *	Total *n* = 222	T1 (*n* = 74)	T2 (*n* = 74)	T3 (*n* = 74)	*p* Value **
MMSE_adj_	27.3 (0.1)	27.8 (0.2)	26.8 (0.3)	26.9 (0.2)	0.003
Age (y)	61.0 (0.4)	60.8 (0.6)	60.9 (0.7)	61.2 (0.6)	0.865
Years from last menstruation (y)	8.8 (0.3)	9.2 (0.5)	8.0 (0.6)	9.0 (0.6)	0.363
BMI (kg/m^2^)	31.9 (0.4)	30.8 (0.6)	32.0 (0.9)	33.2 (0.7)	0.030
Waist circumference (cm)	103.5 (0.8)	102.9 (1.2)	103.6 (1.7)	104.4 (1.1)	0.692
Fat mass (%)	44.3 (0.4)	43.2 (0.6)	44.6 (0.9)	45.6 (0.5)	0.022
Trunk fat mass (kg)	18.9 (0.4)	18.1 (0.6)	19.6 (1.0)	19.5 (0.5)	0.177
hs-CRP (mg/L)	3.8 (0.3)	3.2 (0.4)	3.6 (0.4)	4.5 (0.5)	0.145
TNFα (pg/mL)	8.3 (0.4)	6.9 (0.5)	8.5 (0.6)	9.8 (0.8)	0.004
IL-6 (pg/mL)	2.7 (0.2)	2.1 (0.2)	2.9 (0.4)	3.1 (0.4)	0.053
Sociodemographic characteristics of the study population					
Education (y)	14.0 (0.2)	14.5 (0.3)	14.0 (0.5)	13.0 (0.3)	0.064
Living alone, yes (*n*,%)	37 (17)	21 (23)	9 (16)	7 (10)	0.087
Currently working, yes (*n*,%)	151 (68)	59 (63)	39 (68)	53 (74)	0.379
Physical activity					
Low < 600 MET/min/wk (*n*,%)	42 (19)	14 (15)	9 (16)	19 (29)	0.079
Moderate 600–1499 MET/min/wk (*n*,%)	160 (72)	71 (76)	39 (68)	50 (69)	
High >1499 MET/min/wk (*n*,%)	20 (9)	8 (9)	9 (16)	3 (4)	

* Continuous data are presented as means and standard errors of mean (SEM), categorical data as *n* (%); BMI: body mass index; E-DII: Energy-adjusted Dietary Inflammatory Index; MET: metabolic equivalent; MMSE_adj_: Mini-Mental State Examination, adjusted for age and years of education; PA: physical activity. Tertile 1 of E-DII—the lowest E-DII scores (anti-inflammatory diet group), tertile 3 of E-DII scores—highest E-DII scores (most proinflammatory group). ** A Chi2 test was performed for categorical variables, and nonparametric ANOVA was used for continuous variables.

**Table 2:** Distribution of study participants by degree of cognitive function.

Parameters *	Cognitive Impairment (≤25 Scores) (*n* = 25)	Normal Cognition (>25 Scores) (*n* = 197)	*p* Value **
E-DII	2.2 (0.3)	1.1 (0.1)	0.001
Age (y)	60.1 (1.2)	61.1 (0.4)	0.363
Years from last menstruation (y)	8.7 (1.1)	8.8 (0.4)	0.931
BMI (kg/m^2^)	32.3 (1.7)	31.8 (0.4)	0.744
Waist circumference (cm)	103.3 (2.6)	103.6 (0.8)	0.899
Fat mass (%)	44.2 (1.3)	44.4 (0.4)	0.896
Trunk fat mass (kg)	18.9 (1.4)	19.0 (0.4)	0.993
hs-CRP (mg/L)	4.2 (1.1)	3.7 (0.3)	0.546
TNFα (pg/mL)	9.4 (1.7)	8.1 (0.34)	0.275
IL-6 (pg/mL)	4.1 (0.8)	2.5 (0.2)	0.004
Sociodemographic characteristics of the study population			
Education (y)	12.7 (0.7)	14.1 (0.2)	0.030
Living alone, yes (*n*,%)	2 (8)	35 (18)	0.217
Currently working, yes (*n*,%)	17 (68)	134 (68)	0.991
Physical activity			
Low < 600 MET/min/wk (*n*,%)	10 (41)	20 (10)	0.001
Moderate 600–1499 MET/min/wk (*n*,%)	13 (52)	147 (75)	
High >1499 MET/min/wk (*n*,%)	2 (7)	30 (15)	

* Continuous data are presented as means and standard errors of mean (SEM), categorical data as *n* (%); BMI: body mass index; E-DII: Energy-adjusted Dietary Inflammatory Index; MET: metabolic equivalent; MMSEadj: Mini-Mental State Examination, adjusted for age and years of education; PA: physical activity. ** A Chi2 test was performed for categorical variables, and the Mann–Whitney *U* test for continuous variables.

**Table 3:** Odds ratios for cognitive impairment by E-DII tertiles among postmenopausal women.

E-DII Tertiles	Cognitive Impairment			
	OR ^a^	95% CI	Adjusted OR ^b^	95% CI
Tertile 1 (most anti-inflammatory)	1.00	(Reference)	1.00	(Reference)
Tertile 2	5.16	(1.09; 24.24)	5.39	(1.05; 27.59)
Tertile 3 (most proinflammatory)	9.05	(2.02; 40.52)	11.10	(2.22; 55.56)
*P* for trend	<0.001		0.002	
E-DII Continuous	1.43	(1.14; 1.79)	1.55	(1.19; 2.02)
*P* for trend	<0.001		0.003	

CI: confidence interval; E-DII: Energy-Adjusted Dietary Inflammatory Index; OR: odds ratio. ^a^ Unadjusted model. ^b^ Adjusted for age and BMI, years of education (continuous), PA (categorical). Tests of linear trend cognitive impairment and E-DII score were calculated by assigning the median value of each tertile to each participant in the tertile, and these values were input into the model as ordinal values.

## Data Availability

The data presented in this study are available on request from the corresponding author.
